# Mass drug administration of praziquantel lowers the susceptibility of school-aged children to *Schistosoma mansoni* in endemic areas

**DOI:** 10.3389/fimmu.2026.1746003

**Published:** 2026-02-25

**Authors:** Justin Komguep Nono, Bernard Marie Bitye Zambo, Mireille Kameni Poumeni, Etienne Borel Soh Bayeck, Leonel Meyo Kamguia, Marie Madeleine Noubissi Fotseu, Vincent Brice Owona Ayissi, Thomas Spangenberg, Claudia Demarta-Gatsi

**Affiliations:** 1 Unit of Immunobiology and Helminth Infections, Laboratory of Molecular Biology and Biotechnology, Institute of Medical Research and Medicinal Plant Studies, Ministry of Scientific Research and Innovation, Yaoundé, Cameroon; 2 Division of Immunology, Faculty of Health Sciences, University of Cape Town, Cape Town, South Africa; 3 Department of Microbiology and Parasitology, University of Bamenda, Bambili, Cameroon; 4 Department of Biochemistry, Faculty of Science, University of Yaoundé 1, Yaoundé, Cameroon; 5 Ecole Doctorale Regionale (EDR) d’Afrique Centrale en Infectiologie Tropicale, Université des Sciences et Techniques de Masuku (USTM), Franceville, Gabon; 6 School of Health Sciences, Catholic University of Central Africa, Yaoundé, Cameroon; 7 Global Health Research and Development of the Healthcare Business of Merck KGaA, Darmstadt, Germany, Ares Trading S.A. (An Affiliate of Merck KGaA, Darmstadt, Germany), Eysins, Switzerland

**Keywords:** mass drug administration, praziquantel, protective immunity, schistosomiasis, school-aged children

## Abstract

**Background:**

Schistosomiasis remains a significant public health challenge in endemic regions, leading to substantial morbidity. While regular mass drug administration (MDA) of praziquantel (PZQ) is a cornerstone schistosomiasis control programs in endemic areas, emerging evidence suggests that its benefits may extend beyond mere parasite killing. we sought to determine whether sustained PZQ MDA promotes the development of protective immunity in school-aged children. Building on previous observations in animal models where repeated cycles of *S. mansoni* infection followed by PZQ treatment enhanced host resistance to reinfection, we hypothesized that repeated MDA of PZQ in endemic settings similarly promotes the development of protective anti-schistosome immunity. Accordingly, this study aimed to translate these observations into real-world evidence and investigate the broader association between regular PZQ administration on schistosomiasis infections, burden dynamics, and associated health outcomes in SAC.

**Methods:**

We performed a cross-sectional study on previously collected samples from school-aged children in schistosomiasis-endemic regions who received repeated MDA of PZQ. Levels of plasma antibodies and cytokines were measured by ELISA.

**Results:**

Analysis of previously collected samples and data from cumulative annual rounds of PZQ treatment demonstrated that regular administration significantly reduced the odds of elevated parasite burdens upon reinfection (AOR = 0.16, 95% CI = 0.01-0.61), and improved hemoglobin levels (AOR = 2.58, 95% CI = 1.22-8.05) and academic performance (AOR = 2.39, 95% CI = 1.11-7.09) in SAC. However, it did not significantly reduce the likelihood of liver fibrosis (AOR = 1.73, 95% CI = 0.45-14.53). Mechanistically, repeated PZQ treatment of SAC was associated with heightened arginine/proline metabolism that translated into higher protective IgE levels (p = 0.002) and increased type-2 cytokine production.

**Conclusion:**

Our study highlights a previously underappreciated advantage of sustained PZQ treatment in SAC from schistosomiasis-endemic areas. Regular deworming with PZQ may rapidly rewire the host to foster the development of protective immune responses, mitigating the risks of heavy reinfection and its sequelae, underscoring the overlooked benefits of PZQ treatment for integrated public health strategies against schistosomiasis.

## Introduction

Schistosomiasis is a leading neglected tropical disease, affecting over 250 million people globally, with nearly 800 million at risk (1, 2). *Schistosoma mansoni*, prevalent in sub-Saharan Africa, causes most intestinal cases and contributes significantly to regional disease burden. Infection begins when adult female worms deposit eggs in the intestine and hepatic vasculature, many of these eggs end up been trapped in host tissues causing granulomatous inflammation and progressive fibrosis (1, 3–5). Blood loss and anemia result from chronic gastrointestinal bleeding and immune responses to the parasite, which can lead to severe pathology, including tissue fibrosis, portal hypertension, hepatosplenomegaly, and esophageal varices (1, 3–5), which may be fatal if untreated. To control schistosomiasis, the World Health Organization recommends regular mass treatment cycles of praziquantel (PZQ) in endemic regions to reduce infection burden (2, 6–10). While PZQ mass drug administration programs have had successes, further strategic refinement is needed for optimal disease management (2, 6, 10, 11). This calls for careful evaluation of the impact of regular PZQ MDA in endemic areas.

The development of immunity to Schistosome infections, characterized by a progressive humoral response from repeated exposure to parasite antigens during worm senescence and death (12–14), has attracted significant research interest in endemic populations. Additionally, repeated praziquantel (PZQ) mass drug administration (MDA) in these areas appears to be associated with modulation of host immune response, likely due to antigens exposure released by dying worms (15–22).

Studies show that PZQ-induced death of schistosome worms causes both short-term and long-term changes to the host immune system (23–25) by disrupting the worm protective tegument and exposing hidden parasite antigens. This exposure triggers increased cytokine production (IL-4 and IL-5) and enhances IgE antibody responses (16, 19, 20, 25–27), though the exact mechanisms of this protective immunity remain unclear.

Our group previously found that repeated cycles of *S. mansoni* infection followed by PZQ treatment enhanced host resistance to reinfection in mice (28). However, whether these observations translate to human exposed to repeated MDA of PZQ in endemic settings remains poorly defined. Notably, few studies have examined the long-term immunological effects of repeated PZQ MDA in school-aged children, or its concurrent effects on reinfection risk, morbidity, and fibrosis-related outcomes (16, 26, 29). Building on these gaps, we assess whether prolonged PZQ MDA encourages the development of protective anti-schistosome immunity beyond temporary parasite clearance using human, field-based data from schistosomiasis-endemic areas. We specifically examine how repeated PZQ mass drug administration (MDA) in school-aged children influences reinfection risk, morbidity, and broader immunological benefits beyond just transient infection control.

## Materials and methods

### Ethics statement

Ethical approvals (N°2018/02/976/CE/CNERSH/SP; No2021/12/1417/CE/CNERSH/SP and No2022/12/1505/CE/CNERSH/SP) from the National Ethics Committee of Cameroon and administrative authorizations of research (AAR No631-12.18; prolongation D52.22/L/MINSANTE/DROS/DROS/CRSPE/CEA2 were obtained for the original cross-sectional ‘Maquisard’ study (30) and renewed under approval D12.23/L/MINSANTE/SG/DROS/CRSPE/BBM) from the Ministry of Public Health of Cameroon for this study using archived data and samples. Written informed consents and assents were secured from school children and their legal guardians for the original study and subsequent sample use.

### Study population and participants

We utilized archived data and samples from the *Maquisard* study, a clinical survey of SAC from schistosomiasis-endemic areas with documented PZQ MDA history, infection status, and biological samples (30).

The Maquisard study, conducted five months after the most recent PZQ MDA, was a cross-sectional survey carried out in five public schools located near schistosome-infested rivers across five villages namely: Bongando, Ediolomo, Kedia, Yoro 1, and Yoro 2, within the Bokito subdivision of the Centre Region of Cameroon (**Supplementary Figure 1**). Children were eligible if they had resided for at least six months in the endemic area, were in apparent good health based on clinical examination by the research team and attended one of the selected schools at the time of data collection. All five schools within the same community are located within a 10 km radius and fall under the same Bafia health district, which implements a uniform MDA schedule coordinated by the Ministry of Public Health and supervised by the Cameroon National Program for the Control of Schistosomiasis and Soil-Transmitted Helminthiasis (PNLSHI). As a result, SAC across these sites were exposed to similar treatment schedules and frequencies, minimizing the likelihood of school-level differences influencing treatment exposure or transmission force thus infection intensity. The study was conducted five months after the most recent PZQ MDA. In this setting, PZQ MDA occurs annually, meaning any additional treatment rounds beyond the most recent one took place over 12 months earlier. Consequently, all participants were assessed at a consistent post-treatment interval, ensuring that earlier treatment rounds are unlikely to introduce systematic short-term differences in the analyses (31).

**Figure 1:**
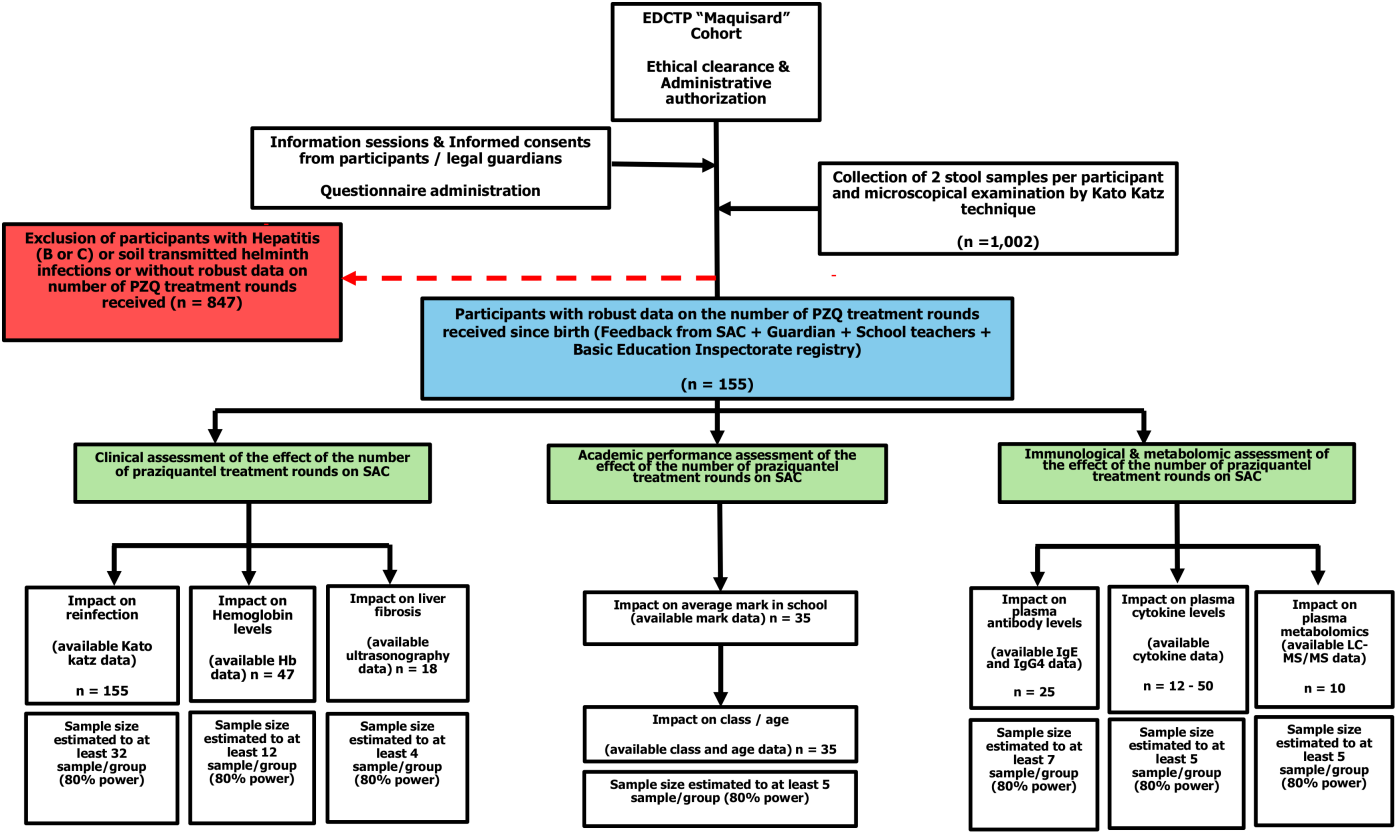
Flow diagram describing the enrolment strategy, examination process and design of participant groups for each experimental query. For the selection, data from SAC enrolled during the *Maquisard* study, with provided consent/assent, free from concomitant infections with hepatitis B or C or geohelminths and presenting with a robust record of their cumulative annual rounds of PZQ treatment received, were selected and their samples prepared for downstream analyses. Infection was defined as the presence of *S. mansoni* eggs in the participant’s stool, as observed by Kato Katz (KK) technique; Liver fibrosis was defined using the liver image pattern (LIP) score. For clinical assessment, participants with PZQ history data were analyzed for the likelihood of having a *S. mansoni* infection, as judged by Kato Katz (n=155); probed for the variation in their hemoglobin levels (n = 47) or presence of liver fibrosis (n=18). Academic performance depending on the number of rounds of PZQ received was also assessed testing the average mark of participants depending on their PZQ rounds numbers (n = 35) and seeking for supporting evidence by comparing the class over age ratios in this population (n=35). Finally, immunological and metabolomics assessment of the changes that might be due to the differential numbers of PZQ rounds of treatment received by participants was done by either checking the dynamics of the plasma IgE and IgG4 levels (n=25); the plasma cytokine levels for GM-CSF, IL-2, IL-33, IL-4 and VEGF (n=12-50) or plasma metabolomic changes observed after LC-MS/MS-based untargeted sequencing (n=10). Minimum sample size for each experimental query is provided to ensure a minimum power of 80% in these analyses in order to reliably unveil differences in the observed outcomes depending on the cumulative number of rounds of PZQ received. KK: Kato-Katz, *Maquisard*: an EDCTP and UK Royal Society funded study on school children from rural Cameroon (30, 32).

In the present study, convenient selection of participants was done as described (**Figure 1**). Participants were selected based on availability of data on cumulative PZQ rounds. Next, all participants presented with infection status making it the most complete group that encloses all other sub-groups used for other downstream analyses. For each of the subsequent assessment done, the availability of the necessary sample and/or dataset determined which of the SAC was included or not e.g. Metabolomics would require plasma samples sent for metabolomics processing and LC-MS-MS analysis, School performance would require data on school performances in school registries, hemoglobin would require data on hemoglobin measurement from fresh blood. As such, participants for every analysis might not be the same, as they did not all have the same datasets, stored sample types/quality or tractable information from given registries. As such we had sometimes identical or sometimes just overlapping candidates from one analysis stream to another, but all selected participants did share a ground similarity of having a well-defined number of praziquantel rounds received and a clear link of that to the infectious status known – as this (PZQ treatment history on susceptibility to infection) was the central premise of all subsequent analyses. In summary, infection levels, hemoglobin, liver fibrosis status, academic performance, and sufficient stored plasma to assess the impact of different PZQ administration rounds on clinical (reinfection rates and burden, hemoglobin levels and liver fibrosis status), academic (average mark in school and class/age ratio), immunological, and metabolomic outcomes (plasma IgE/IgG4 levels, cytokine levels and metabolite levels) were used to select participants for each analysis stream. Participants with hepatitis (B or C) or soil transmitted helminths infection or without robust data on the number of PZQ treatment rounds received were the main criteria of non-inclusion.

### Sample size calculations

Sample sizes calculations were performed to compare the effect of different PZQ administration rounds on relevant outcomes such as infection assessment, the onset of liver fibrosis, hemoglobin levels, academic performance, plasma antibodies, and cytokines (**Figure 1**). Calculations were done separately for binary and continuous variables using standard statistical formulas, informed by relevant outcomes reported in previous studies (33–38). A matching approach (age, gender, exposure based) was applied to strengthen the study design by enhancing efficiency, increasing statistical power, and minimizing potential confounding. This ensured a more accurate assessment of associations between cumulative PZQ exposure and the measured outcomes (39).

For binary variables such as infection assessment and liver fibrosis, the required sample size per group was estimated using the formula:


$$n=\frac{{\left(\text{Zα}{/}^{2}\;+\text{Zβ}\right)}^{2}•\left[\text{P}1\left(1-\text{P}1\right)+P2\left(1-P2\right)\right]}{{\left(\text{P}1-\text{P}2\right)}^{2}}$$

where P_1_​ and P_2_​ are the estimated prevalences before and after treatment, respectively. Calculations assumed a two-sided significance level of 5% (α = 0.05, Z_α/2_ = 1.96Z) and 80% power (Z_β_=0.84Z). Minimum sample sizes per group were: 119 for infection assessment and 26 for liver fibrosis (30, 40).

For continuous variables such as hemoglobin levels, plasma antibodies, cytokines, metabolomes, and academic performance, sample sizes were estimated using the following formula (41):


$$n=\frac{{\left(\text{Zα}{/}^{2}\;+\text{Zβ}\right)}^{2}•\;2{\text{δ}}^{2}}{{\text{d}}^{2}}$$

where δ is the standard deviation and d the minimum detectable difference. Calculations used α = 0.05 and power = 80%.

Minimum sample sizes per group were: 25 for hemoglobin levels, 14 for academic performance, 16 for plasma antibodies, and 14 for cytokines (42, 43). To determine the minimum sample size for metabolome comparisons, Cohen’s d effect size was calculated in R as the mean difference between groups divided by the pooled standard deviation. Based on these calculations, the minimum sample size required to detect a meaningful difference was approximately 6 participants per group.

All participants with required data and enough plasma samples were included in analyses, irrespective of providing a enough required samples. Sub-powered comparisons were potentiated by bias removal using only matched participants, as reported (39).

### Exploitation of archived data

#### Sociodemographic data

Archived data from selected participants were used for comparative analyses, with sociodemographic data i.e. gender, age, body mass index (through height and weight measurements) and water contact frequency (assessed through a standardized questionnaire administered at the time of sampling, which captured the frequency and type of activities involving potential exposure to infested water e.g., bathing, swimming, washing clothes, and collecting water) obtained through interviews and three distinct levels of verification i.e. with SAC, parents/guardians, and teachers from the original *Maquisard* study (30, 32). The selected factors were chosen based on their recognized potential as risk factors for infection, infection progression and hepatic fibrosis (HF) (1, 30, 44–46). This matching of risk factors among clusters aimed to mitigate the influence of other potential biases when studying the role of repeated PZQ administration on infection and associated pathology.

#### History of PZQ rounds received

Previous annual PZQ treatments were obtained through a two-step validation process. First, treatment histories were reported by participants and verified by their parents/guardians. Second, to ensure accuracy, these data were cross-verified against formal documentation of school-based MDA activities, including treatment dates, extracted from the basic education inspectorate registries under the Ministry of Basic Education (MINEDUB), which maintain official records of PZQ distribution in participating schools. SAC with incomplete or inconsistent treatment histories or those who had studied outside the study area were excluded to minimize misclassification bias.

Participants were classified by cumulative PZQ exposure (0–2 *vs*. >2 rounds), a threshold guided by preclinical evidence (28).

#### Academic performance indicators

To evaluate cognitive and academic performance, quarterly marks were obtained from the teacher’s class register. Each child’s ratio of age over class was also used as a cognitive performance index (47), with classes recoded into numeric scales from 1 to 6 and the ratio calculated against the child’s age. Since the age/class ratio may be influenced by parents’ participation and baseline cognition, its interpretation becomes of greater significance when considering absolute grades, as it offers important information about how students are progressing academically in relation to their age (48).

#### Parasitological data

Parasitological data on *S mansoni* infection status (egg presence and egg burden) reported during the initial ‘*Maquisard*’ study, as previously described (30, 32). Briefly, two stool samples were collected from each participant within a five-day interval. From each sample, two Kato-Katz (KK) thick smears were prepared and independently examined under optical microscopy (Leica Microsystems, DM2000, Germany) at 10X and 40X magnifications for the detection and quantification of parasites eggs. The smears were read by two experienced laboratory technicians from the Ministry of Scientific Research and Innovation (MINRESI), Yaoundé, Cameroon. The arithmetic mean of eggs across the four KK smears per participant was used to determine individual infection intensity. For quality control, 20 randomly selected slides were re-examined by a third independent laboratory technician to ensure consistency.

Parasitological data on *S mansoni* infection status were subsequently extracted from the *Maquisard* study database and used as defining variables in the comparative analysis assessing the impact of prior PZQ treatment history on reinfection.

#### Ultrasonographic examination records

Liver fibrosis status was determined by abdominal ultrasound examination using portable ultrasound with convex transducer (2–7 MHz) according to WHO guidelines for *S. mansoni* morbidity assessment. Liver Image Patterns (LIP) grades C-F were recorded as positive for liver fibrosis, while LIP grade A was considered negative (30, 32).

#### Blood samples and testing

Archived blood-related laboratory analyses result previously obtained as per established protocols during the ‘*Maquisard*’ study (30, 32) were also extracted for the participants selected in the present study as per **Figure 1**, with exploitation of the following parameters:

a. *Hemoglobin levels*

Hemoglobin levels, recorded using the Tallquist Hemoglobin Scale following the manufacturer’s instructions (49), i.e. visually assessed sequentially by two technicians to avoid errors with validation by a third technicians in case of discordance between the first two observations, were computed as a defining variable for participants selected for the comparison scheme of impact of PZQ treatment history on hemoglobin levels.

b. *Hepatitis B and C screening*

Records of blood testing for hepatitis B and hepatitis C infections (using DiaSpot HBsAg and DiaSpot HCV Ab rapid diagnostic test strip [DIASPOT™ Diagnostic, Indonesia (30)], were used to exclude positive participants from the present analyses.

### ELISA and metabolomic assays on retrieved plasma samples

Archived plasma samples from selected participants of all immunological and metabolomic comparison scheme analyses, obtained from centrifugation of collected blood samples during the *Maquisard* study and kept frozen at-80°C until the present use, were thawed and subjected to serological assessments:

#### Total IgE and IgG4 quantification

Total Human IgE and IgG4 concentrations were quantified in 25 plasma samples each using commercially available ELISA kits following the manufacturer’s protocol. Total IgE levels were measured using Human IgE ELISA kit (BIOMATIK; Catalog Number: EKC31252), and total IgG4 levels were measured using Human IgG4 ELISA kit (BIOMATIK; Catalog Number: EKE61042). All reagents used were provided in the respective kits. Blank wells containing assay diluent and distilled water were used to assess background signal. A matrix control of heat-inactivated and sterile-filtered human plasma was used as matrix control and spiked with standards to ensure adequate performance of kits. Standards were run in parallel with plasma samples under identical experimental conditions, and antibody concentrations were calculated from standard curves. The assay sensitivities were 3.64 ng/mL for total IgE and 0.38 µg/mL for total IgG4.

#### Cytokine quantification

Plasma cytokine concentrations were quantified using commercially available s Sandwich ELISA Kits according to the manufacturers’ protocol. 12 plasma samples was used to measure Human GM-CSF (RayBiotech; LOT: 1009200267), IL-4 (Proteintech, Barcode: 20005524, Cat No: KE00034), IL-2 (Proteintech, Barcode: 40001524, Cat No: KE00017), and VEGF (Proteintech, Barcode: 40001452, Cat No: KE00216). A total of 49 plasma samples was used to measure IL-33 concentrations using the IL-33 Sandwich ELISA Kit (BioLegend, Inc. USA Cat No: 435907) following the manufacturers’ protocol.

For all assays, plasma samples were diluted 1:1 in the provided assay diluent. All reagents were supplied with the respective kits. Blank wells containing assay diluent and distilled water were used to assess background signal. A matrix control of heat-inactivated and sterile-filtered human plasma was used as matrix control and spiked with standards to ensure adequate performance of kits. Standards were run in parallel with plasma samples under identical experimental conditions, and cytokine concentrations were calculated from standard curves. The limits of detection were 2 pg/mL for GM-CSF, 0.1 pg/mL for IL-4, 3.6 pg/mL for IL-2, 1 pg/mL for VEGF, and 4,14 pg/ml for IL-33.

#### Plasma metabolomics

Selected plasma samples underwent LC-MS analysis using ZIC-pHILIC chromatography with Q Exactive HF mass spectrometer to collect positive and negative ion data. The Polyomics integrated Metabolomics Pipeline (PiMP) was used for data preprocessing, including peak detection, alignment, normalization, and metabolite annotation.

### Statistical analysis

Data were entered in Excel, cross-checked for errors, and analyzed using RStudio (version 4.3.1) with graphs plotted in GraphPad Prism 8 (version 10.1.2.324). Statistical analyses included descriptive measures, normality testing (Shapiro-Wilk), group comparisons (t-tests, Mann-Whitney, ANOVA, Kruskal-Wallis), and correlations (Pearson, Spearman). Multivariate logistic regression assessed the predictive potential of cumulative PZQ rounds on various outcomes, including *S. mansoni* infection (positive KK thick smear), higher infection burden (greater than 100 EPG of feces), higher hemoglobin levels (greater or equal to 11 g/dL), onset of liver fibrosis in infected individuals, good academic performance (average mark ≥ 12), and good academic progression (class/age ≥ 0.50). Most of these variables were analyzed by dichotomizing of the data sets to ensure relevance for real-world outcomes (i.e. anemic or not, fibrotic or not, academically above average or not etc.) and avoid comparison on a continuous basis that might reveal non-relevant differences for real-world settings. For all analyses, a p-value< 0.05 was considered statistically significant and the specific statistical test used is mentioned in the corresponding figure legend.

For metabolomic analysis, data processed by PiMP were exported in CSV format to MetaboAnalyst version 6.0 for statistical analysis. To improve the normality of the data distribution, data was transformed and scaled using respectively Logarithmic transformation (log10) and Pareto Scaling. Variable importance in projection (VIP) scores of the first two principal components in multivariate PLS-DA model, combined with fold change (FC) and p-value of univariate analysis were considered as filters to select differentially expressed. The filters were: 1) VIP ≥ 1.5; 2) fold-change ≥ 1.5 or ≤ 0.6667 and 3) p-value< 0.05. All three conditions were strictly needed for a metabolite feature to be considered as a differential feature. Only human-derived features were selected from the list of differential metabolites features obtained to perform a metabolic pathway analysis based on the KEGG database.

## Results

### Demographic characteristics of enrolled participants

The study enrolled school-aged children (5–18 years) five months after their most recent PZQ treatment (30). SAC were categorized into two groups based on treatment history (0–2 PZQ rounds *vs >*2 PZQ rounds) (**Table 1**) as informed by prior murine preclinical study suggesting enhanced resistance following multiple treatments (28). While demographic characteristics were largely comparable between groups, children with >2 PZQ treatments were significantly older (p=0.01), which was adjusted for in subsequent analyses to ensure fair comparison of treatment outcomes.

**Table 1:** Social demographic characteristics of the selected participants across the different PZQ treatment groups for each experimental query.

Analysis subset	Variables	PZQ groups		p-value
		0–2 PZQ	>2 PZQ	
Impact on infection: available Kato Katz data (n = 155)	Age in years: Mean (SD)	9.23 (1.79)	10.00 (1.66)	**0.01**
	Sex ratio (Male/Female)	68/44	23/20	0.46
	BMI: Mean (SD)	15.31 (1.37)	15.67 (1.29)	0.15
	Frequency of contact with water/day:Mean (SD)	1.85 (0.76)	1.93 (0.73)	0.57
Impact on Hemoglobin levels (available Hemoglobin data) n = 47	Age in years: Mean (SD)	9.86 (1.48)	10.17 (1.65)	0.67
	Sex ratio (Male/Female)	18/11	8/10	0.68
	BMI: Mean (SD)	15.85 (1.44)	15.96 (1.05)	0.91
	Frequency of contact with water/day:Mean (SD)	2.04 (0.90)	1.72 (0.66)	0.13
Impact on liver fibrosis (available ultrasonography data) n = 18	Age in years: Mean (SD)	10.36 (2.02)	12.25 (2.36)	0.12
	Sex ratio (Male/Female)	8/6	3/1	>0.99
	BMI: Mean (SD)	16.17 (1.46)	17.00 (1.59)	0.34
	Frequency of contact with water/day: Mean (SD)	2.14 (0.86)	1.75 (0.95)	0.44
Impact on average mark and class/age in school (available mark and class data) n = 35	Age in years: Mean (SD)	9.81 (2.05)	9.75 (1.98)	0.93
	Sex ratio (Male/Female)	17/10	4/4	0.68
	BMI: Mean (SD)	15.85 (1.63)	15.78 (1.86)	0.91
	Frequency of contact with water/day:Mean (SD)	2.29 (0.91)	2.00 (0.92)	0.44
Impact on plasma antibody levels (available IgE and IgG4 data) n = 25	Age in years: Mean (SD)	10.28 (2.36)	10.22 (1.62)	0.91
	Sex ratio (Male/Female)	3/4	9/9	0.90
	BMI: Mean (SD)	16.07 (1.65)	16.01 (1.05)	0.82
	Frequency of contact with water/day:Mean (SD)	2.28 (0.95)	1.77 (0.64)	0.20
Impact on plasma cytokine levels (available cytokine data) n = 12	Age in years: Mean (SD)	10.00 (2.44)	10.83 (1.47)	0.49
	Sex ratio (Male/Female)	3/3	5/1	0.54
	BMI: Mean (SD)	16.16 (1.79)	16.34 (1.30)	0.84
	Frequency of contact with water/day:Mean (SD)	2.50 (0.83)	2.00 (0.89)	0.34
Impact on plasma metabolomics (available LC-MS/MS data) n = 10	Age in years: Mean (SD)	10.17 (2.31)	10.00 (1.82)	0.90
	Sex ratio (Male/Female)	4/2	2/2	>0.99
	BMI: Mean (SD)	16.26 (1.73)	16.14 (0.44)	0.90
	Frequency of contact with water/day:Mean (SD)	2.33 (1.03)	1.75 (0.95)	0.39

SD, Standard Deviation; BMI, Body Mass Index; n, number of participants; p, p-value.

0–2 PZQ (n) or > 2 PZQ (n); Both non-parametric and parametric testing were performed yielding similar outcomes. Mann-Whitney-U test and Chi-square test results are displayed. The bold values indicate statistically significant p-values.

### Multiple PZQ rounds reduce high-burden infections and anemia but not liver fibrosis

**Figure 2:**
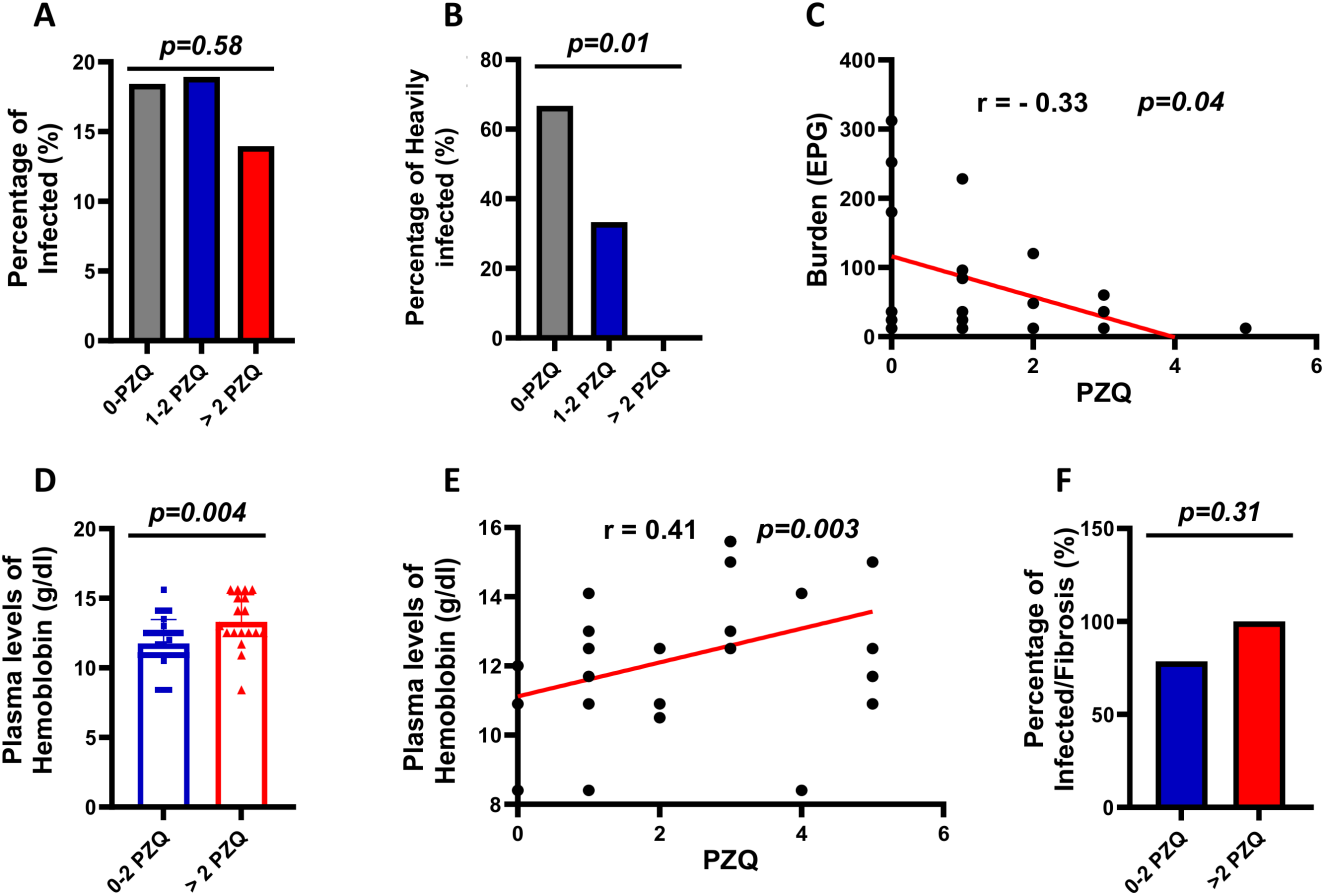
Clinical phenotyping of SAC from a schistosomiasis endemic area based on different numbers of annual PZQ rounds. **(A)** Frequencies of *S. mansoni* infections across participants with increasing treatment rounds (n=155). **(B)** Frequencies of high-burden *S. mansoni* infections (≥100 EPG) across participants (n=27). **(C)** Spearman correlation between PZQ treatment numbers and egg burden in infected participants (n=27). **(D)** Average hemoglobin levels across increasing treatment rounds (n= 47). **(E)** Spearman correlation between PZQ treatments and hemoglobin levels (n= 42). **(F)** Frequencies of liver fibrosis among *S. mansoni*-infected participants across treatment rounds (n = 18). Statistical analyses: Chi-square test for trend **(A, B, F)**, Spearman correlation with coefficient (r) and p-value **(C, E)**, and non-parametric Mann-Whitney U test **(D)**. Red regression lines illustrate overall trends in correlation plots. P< 0.05 is considered the difference to be statistically significant. EPG, Egg Per Gram of feces.

To evaluate the clinical impact of repeated annual PZQ administration, we assessed *S. mansoni* infection prevalence, parasite burdens (eggs per gram of feces), hemoglobin levels, and liver fibrosis in our selected participants (**Figure 2**). Multiple annual PZQ rounds did not reduce *S. mansoni* infection prevalence (p = 0.58; **Figure 2A**) but significantly reduced high-burden infections (p = 0.01; **Figure 2B**). This was specifically apparent in children that had received more than 2 rounds of treatments (**Figures 2, B**). All subsequent analyses were therefore done using the specific clustering of participants into those that had received a maximum of 2 rounds of PZQ in their lives and those that had received more than 2 rounds of PZQ i.e. >PZQ. With this clustering, similar to that used in our preclinical study (28), we observed increased hemoglobin levels (p = 0.004; **Figure 2D**), with no impact on liver fibrosis prevalence for SAC with higher rounds of PZQ received i.e. >2PZQ when compared to those with a history of less cumulative rounds of PZQ i.e. 0–2 PZQ. Multivariate analysis confirmed that higher cumulative PZQ treatments were associated with reduced likelihood of high-burden infection (AOR = 0.16; p = 0.03) and increased odds of elevated hemoglobin (AOR = 2.58; p = 0.03), but not with reinfection risk or liver fibrosis development (**Table 2**).

**Table 2:** Risk factors for *S. mansoni* infection, high burden, and fibrosis.

Factors	Clinical phenotype assessment of the effect of the number of PZQ rounds on SAC							
	Predicted probability							
	Being infected		Being heavily infected		Higher hemoglobin		Being infected with liver fibrosis	
	AOR (95% CI)	p-value	AOR (95% CI)	p-value	AOR (95% CI)	p-value	AOR (95% CI)	p-value
Number of PZQ	0.76 (0.53-1.04)	p=0.11	0.16 (0.01-0.61)	**p=0.03**	2.58 (1.22-8.05)	**p=0.03**	1,73 (0.45-14.53)	p=0.46
Age in years	1.31 (1.00-1.74)	p=0.05	1.25 (0.50-3.45)	p=0.61	1.88 (0.95-5.16)	p=0.10	0.91 (0.34-2.68)	p=0.86
Gender (Male)	1.15 (0.45-3.04)	p=0.77	1.21 (0.05-23.58)	p=0.89	0.79 (0.11-4.72)	p=0.80	0.28 (0.05-5.61)	p=0.42
Frequency of contacts with water/Day	0.69 (0.37-1.27)	p=0.25	0.25 (0.02-1.26)	p=0.15	0.47 (0.12-1.38)	p=0.20	1.28 (0.21-8.99)	p=0.77
Body Mass index (BMI)	1.38 (0.96-2.05)	p=0.09	2.24 (0.73-11.35)	p=0.22	0.58 (0.24-1.34)	p=0.20	1.36 (0.32-6.89)	p=0.66

Heavily infected, burden higher than 100 EPG; Higher haemoglobin, hemoglobin level higher than 11 g/dl; EPG, Eggs Per Gram; AOR, Adjusted Odds Ratio; 95% CI, 95% Confidence Interval. The bold values indicate statistically significant p-values.

### Multiple annual rounds of PZQ administration improve SAC cognitive performance

**Figure 3:**
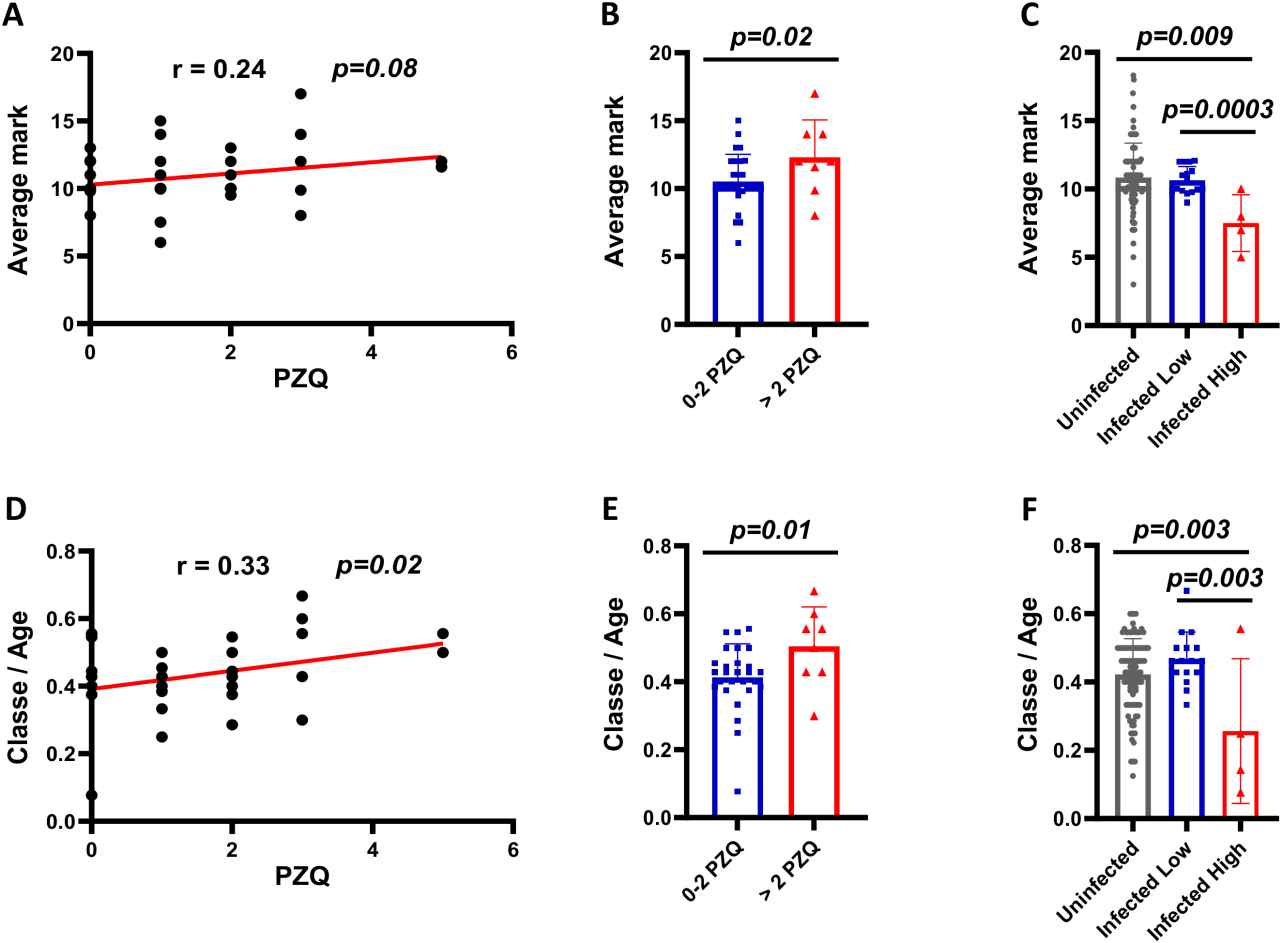
Academic performance of SAC from a schistosomiasis endemic area based on PZQ treatment history. **(A)** Correlation between PZQ treatments and average mark (n= 35). **(B)** Average marks across increasing treatment rounds (n= 35). **(C)** Average marks by *S. mansoni* infection burden (n= 20). **(D)** Correlation between PZQ treatments and class/age ratio (n= 35). **(E)** Class/age ratio across increasing treatment rounds (n= 35). **(F)** Class/age ratio by *S. mansoni* infection burden (n= 35). Statistical analyses: Spearman correlation with correlation coefficient (r) and p-value (p) reported, Mann-Whitney U test for bar graph comparisons. P< 0.05 is considered the difference to be statistically significant. Infection categories: Uninfected (0 EPG), Infected Low (1–99 EPG), Infected High (≥100 EPG). EPG, Eggs per gram of stool.

We assessed academic performance through average school grades and class/age ratios in relation to cumulative PZQ exposure, finding significantly higher achievement and progression in children receiving >2 annual PZQ treatments compared to those with ≤2 rounds (**Figures 3A–F**). Higher egg burdens were associated with lower academic performance, while more PZQ rounds correlated positively with both grades and class/age ratios (**Figures 3A, D**). After adjusting for confounding factors such as age, sex, infection intensity, and exposure risk (approximated by frequency of contact with water), multivariate analysis confirmed that children with >2 PZQ rounds were more likely to show improved academic performance (AOR = 2.39, p=0.05) and progression (AOR = 2.33, p=0.03) (**Table 3**). Because school grades are recognized indicators of cognitive function in children (48), these findings suggest that repeated praziquantel treatment may contribute to improved cognitive performance among school-aged children in endemic areas.

**Table 3:** Assessment of risk factors predisposing to *S. mansoni* cognitive impairment.

Factors	Clinical phenotype assessment of the effect of the number of PZQ rounds on SAC			
	Predicted probability			
	Risk factors of better academic performance (Average mark ≥12)		Risk factors of unimpaired academic progression (Class/Age > 0.50)	
	AOR (95% CI)	p-value	AOR (95% CI)	p-value
Number of PZQ	2.39 (1.11-7.09)	**p=0.05**	2.33 (1.18-6.16)	**p=0.03**
Age in years	0.24 (0.00-0.59)	**p=0.01**	0.80 (0.47-1.30)	p=0.39
Gender (Male)	0.19 (0.018-1.42)	p=0.12	1.13 (0.20-7.34)	p=0.88
Frequency of contacts with water/Day	3.81 (0.93-24.15)	p=0.09	2.42 (0.77-9.16)	p=0.14
Body Mass index (BMI)	1.13 (0.52-2.74)	p=0.69	0.97 (0.51-1.81)	p=0.93

AOR, Adjusted Odd Ratio; 95% CI, 95% Confidence Interval. The bold values indicate statistically significant p-values.

### Regular PZQ administration enhances type-2 and angiogenic responses

**Figure 4:**
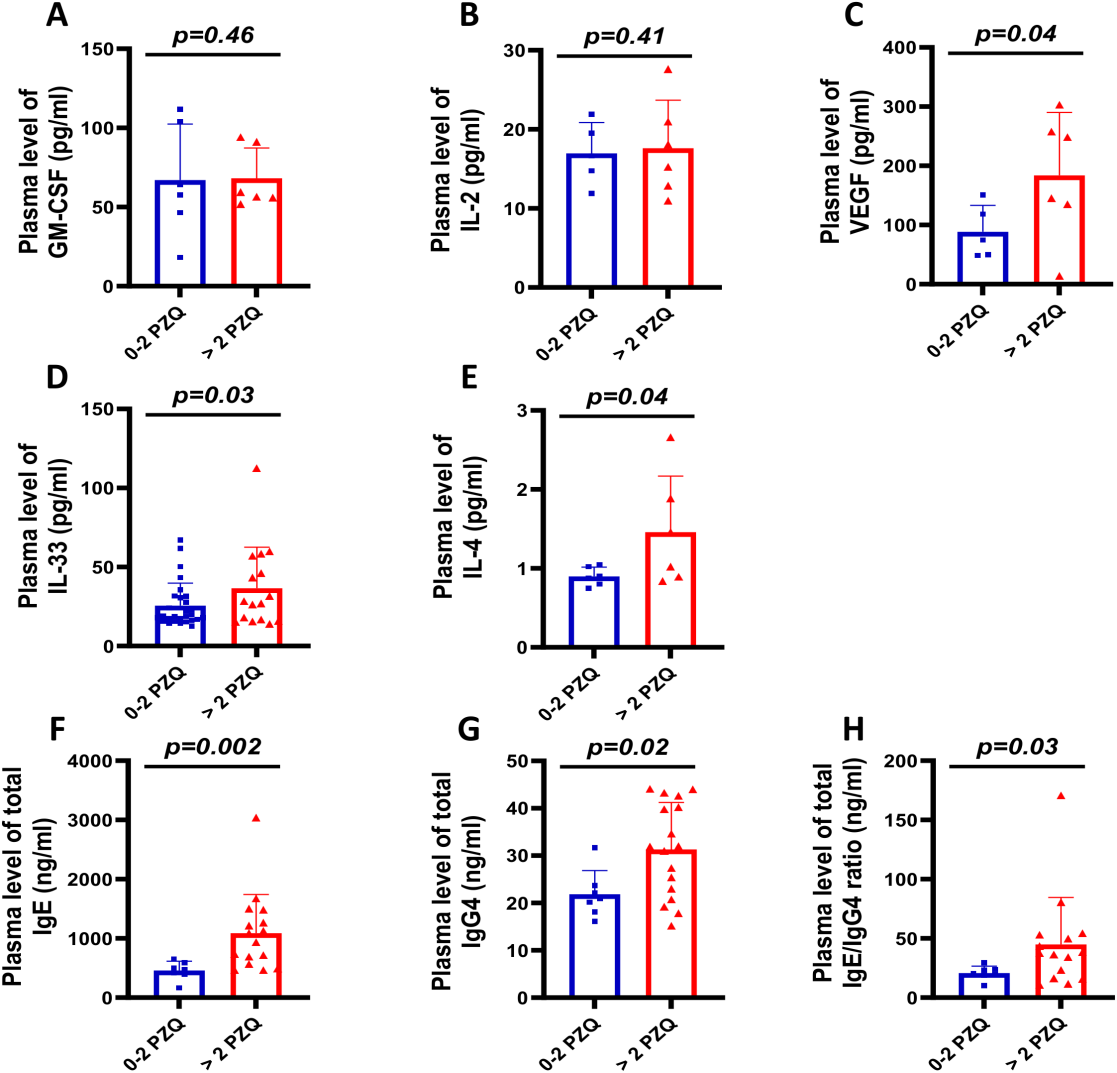
Immunological profiling of SAC across different PZQ rounds. Comparison of plasma levels for: **(A)** GM-CSF (innate myeloid cytokine) (n=12), **(B)** IL-2 (adaptive cytokine) (n=12), and **(C)** VEGF (angiogenic profibrotic factor) (n=12). **(D)** IL-33 levels (alarmin associated with *S. mansoni* infection) (n=49). **(E)** IL-4 (Th2 cytokine) levels (n=12). Immunoglobulin comparisons: **(F)** Total IgE (n=25), **(G)** Total IgG4 (n=25), and **(H)** IgE/IgG4 ratios as reinfection protection indicators (n=25). Graphs represent mean with standard deviation. Statistical analysis: Non-parametric Mann-Whitney U test (significance at p<0.05). GM-CSF, Granulocyte-macrophage colony-stimulating factor; IL, Interleukin; VEGF, Vascular Endothelial Growth Factor.

To investigate the underlying immune responses associated with repeated PZQ exposure (**Figures 4A–H**), we measured plasma levels of canonical immune markers: GM-CSF (50), IL-2 (51), and VEGF (52). We measured plasma levels of immune markers and found no differences in GM-CSF (**Figure 4A**) and IL-2 (**Figure 4B**) between treatment groups, but VEGF levels were elevated in children receiving >2 PZQ rounds (p=0.04) (**Figure 4C**), suggesting increased angiogenic activity. Children with >2 PZQ treatments showed significantly elevated type 2 immunity markers including IL-33 (p=0.03) (**Figure 4D**) and IL-4 (p=0.04) (**Figure 4E**). Total IgE and IgE/IgG4 ratios were also significantly increased in the >2 PZQ group (p=0.002; p=0.03) (**Figure 4H**), indicating enhanced anti-schistosomal immune readiness. No correlations were observed between theses studied cytokines or antibodies and egg burden at the present sample size used for the cytokine assessment stream (n = 12-50)(**Supplementary Figure 2**).

### Regular PZQ administration upregulates arginine/proline pathway

**Figure 5:**
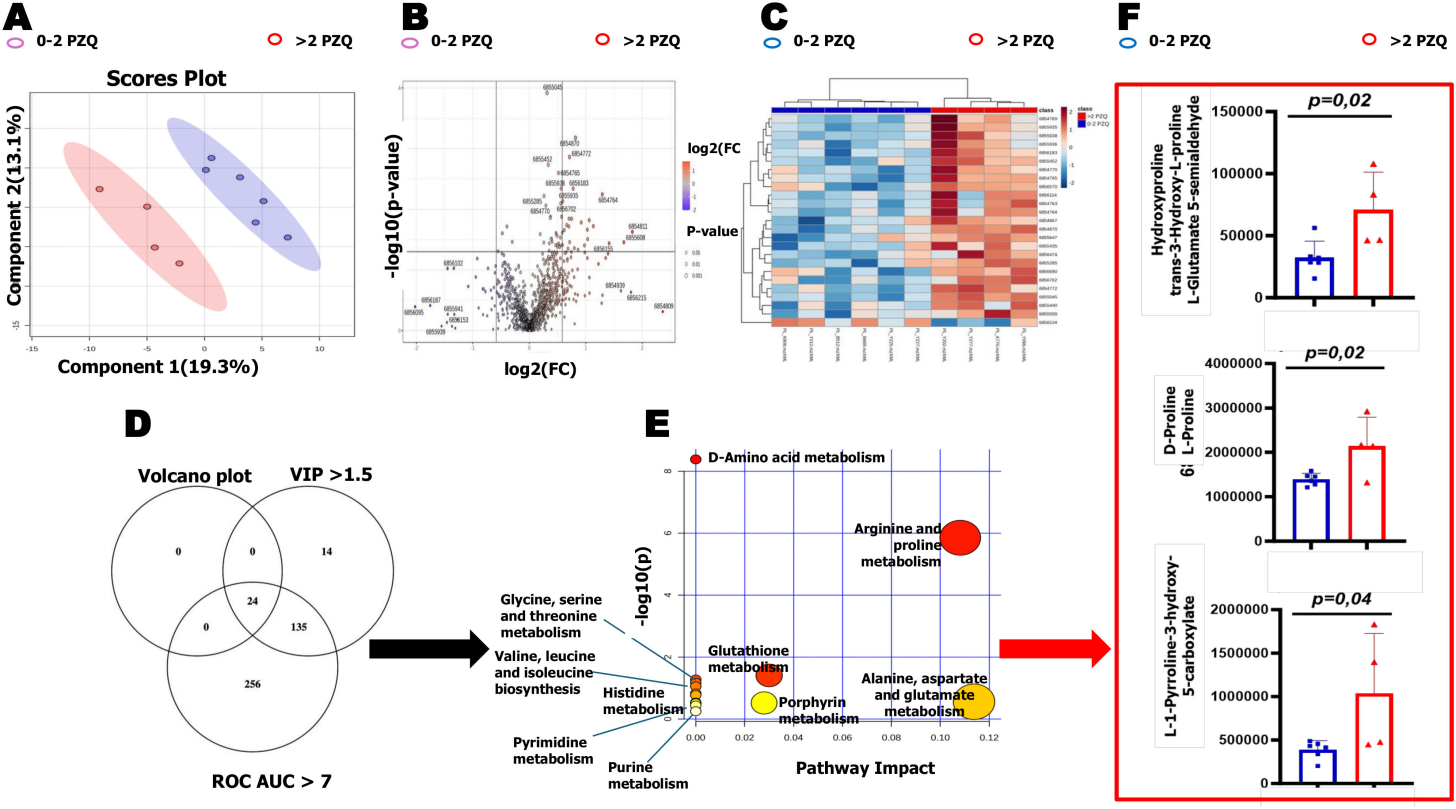
Metabolomic profiling of SAC across different PZQ rounds. **(A)** Partial Least Squares Discriminant Analysis (PLS-DA) score plots distinguishing SAC with ≤2 PZQ treatments (blue) from >2 treatments (red). **(B)** Volcano plot comparing metabolite expression between treatment groups, highlighting differentially expressed metabolites (Fold change ≥1.5, p-value<0.05), with a total of 24 significantly upregulated metabolites compared to downregulated ones; the x-axis represents log_2_ fold change (FC) and the y-axis represents −log_10_(p-value). **(C)** Hierarchical cluster analysis of 25 most significant metabolic features, showing peak burden variations between 0–2 PZQ and >2 PZQ groups. Data normalized, log-transformed, and Pareto-scaled. **(D)** Refined selection of most discriminating metabolites using Venn diagram illustrating overlap between volcano plot significant hits, VIP scores >1.5, and ROC curve analysis (AUC >0.7), yielding 24 metabolites used for pathway analysis. **(E)** Pathway map of affected metabolic pathways generated in MetaboAnalyst, where the y-axis represents −log_10_(p-value) and the x-axis represents pathway impact; circle size reflects pathway impact and color (yellow to red) indicates increasing statistical significance. **(F)** Peak intensities of identified metabolites from arginine/proline metabolism, with multiple peak annotations. Note several PIMP annotations were possible for a single peak ID resulting in 3 peak graphs for the 6 metabolites identified. Visualization highlights metabolic changes associated with increasing PZQ rounds.

Untargeted metabolomic profiling revealed distinct clustering between children receiving 0–2 *vs*. >2 annual PZQ round, as shown by Partial Least Squares Discriminant Analysis (PLS-DA), indicating distinct metabolic signatures associated with repeated treatment (**Figures 5A–C**). Differential metabolite analysis identified 24 metabolites (**Figure 5D**) significantly discriminating between treatment groups. Pathway enrichment analysis identified significant upregulation of the arginine and proline metabolism among the 24 metabolites that significantly discriminated between groups in children exposed to more than (>2) PZQ rounds (**Figure 5E**). All key metabolites within this pathway were consistently elevated in the plasma of children with >2 PZQ treatments (**Figure 5F**), establishing arginine/proline metabolism as a critical molecular signature of repeated PZQ exposure.

## Discussion

Despite the efforts made through annual MDA of PZQ, schistosomiasis remains a significant health problem in tropical regions and PZQ is the mainstay of the global strategies aimed at the elimination of schistosomiasis as a public health problem (9). Repeated PZQ treatments combined with continuous schistosome exposure reduce host susceptibility to reinfection in preclinical settings (28), suggesting PZQ may accelerate protective immunity development beyond its antiparasitic properties (53).

The possibility of PZQ-induced acquired immunity, potentially shaped by past infection and treatment exposure, raises important questions about the relationship between treatment frequency, immune adaptation, and long-term control of schistosomiasis. It emphasizes the need to explore how repeated PZQ administration shapes host immune responses and contributes to lasting reductions in morbidity and transmission.

Our findings indicate that SAC receiving more cumulative PZQ rounds exhibited significantly lower egg burdens five months post treatment, suggesting reduced parasite burden over time with regular treatments in areas of sustained transmission. Although we might have some limitations on the causal link between the improved pro-protective immunity observed and the reduced rate of re/infection, due to the limitations of our study design, these findings are in line with previous preclinical findings (28) and the suggested hypothesis that repeated treatment may eventually affect host–parasite interactions over time towards rendering treated hosts less permissive to infection/infection progression (28, 29). On the possible caveat of persistent infections in some hosts that are more pronounced when treatment rounds are lower, our study design clearly opposes the facts that i) MDA of PZQ in the study area occurs annually, any additional rounds beyond the most recent one took place more than 12 months earlier, making it unlikely that the observed differences reflect short-term pharmacological activity; ii) furthermore, the possibility of treatment variation between groups is reduced because each participant received the same standardized PZQ dose; iii) although the reduced re/infection burden observed may not result from treatment-induced immune sensitization (12, 13), behavioral factors like reduced water contact (54) were controlled and matched between groups as well as other possible confounders such as age, gender and BMI, to ensure as much as feasible that the observed correlation between cumulative PZQ exposure and decreased egg burden remained significant and mostly explainable by differential immune responses; iv) total IgE levels were significantly higher in children who received more PZQ rounds compared to those with less rounds, indicating enhanced type-2 immune activation rather than merely lower parasite exposure at play here. Hence, if reduced egg counts were driven primarily by better long-term drug efficacies in SAC that might have remained infected for long and subjected to more PZQ rounds, total IgE levels would be expected to decline with such successive rounds of treatment due to decreased antigenic stimulation but this is not the case. On the above premises, our findings rather support the hypothesis that an augmented immune-mediated mechanism, rather than behavioral changes alone, or ameliorated clearance in one group over the other group underlies the observed acquired reduced infection burden, likely to align with acquired protection in the present setting (55).

Building on these overall general premises set in our study, and now guided by previous studies (16–20, 23), we advocate for the idea that PZQ-induced worm death will rapidly release antigens that prime host immunity, enhances resistance to future infections and extends the drug relevance beyond transient deworming to promote long-term protection. While overall prevalence did not vary by treatment history, the likelihood of high-burden infections, strongly linked to morbidity (9, 10), was markedly reduced in children receiving repeated PZQ rounds, accompanied by significantly decreased anemia (1, 5), likely due to reduced inflammation and reduced blood loss associated with fewer eggs crossing the gut wall.

Cognitive performance (higher class grades and age-appropriate progression) was significantly better in SAC receiving more cumulative PZQ treatments, aligning with previous observations (35, 56). This improvement is likely related to an association between reduced reinfection burden and improved cognitive outcomes, potentially due to alleviation of physical discomfort from worm presence, anemia, and malnutrition linked to chronic infection (5, 35, 56). This is supported by our observation of declining cognitive performance with increased egg burden. Although the present data does not definitively determine whether enhanced cognitive performance results from anti-parasitic activity or reduced systemic inflammation (35), our findings suggest inadequate PZQ treatment may be associated with poor cognitive performance in SAC living in schistosomiasis-endemic regions.

Our data also revealed elevated type-2 cytokines (IL-33, IL-4) (30) and IgE production in SAC receiving more regularly PZQ, supporting protective immunity against schistosomiasis and correlating with lower egg burdens. Elevated VEGF levels in these extensively treated children suggest a potential trade-off between anti-worm immunity and profibrotic responses (57), as liver fibrosis persisted despite apparent reinfection/infection resistance. This “double-edged sword” scenario, consistent with preclinical observations of progressive fibrosis despite anti-parasite protection, suggests that sustained PZQ treatment may enhance protection against heavy infections while potentially driving stronger fibrotic responses possibly through recognition of cross-reactive egg antigens (58, 59). The definitive assessment of a potential risk of more fibrogenic responses from hosts repeatedly exposed to cycles of infection/treatment is needed and will require larger and longitudinal studies.

As such, however, this possible scenario, already observed in preclinical settings (28) i.e. of acquired fibrogenic reactivity upon cycles of infection/treatment contrasts with previous reports (59) suggesting that PZQ may have anti-fibrotic properties; however, mechanistic studies that distinguished its parasite-killing ability from potential fibrosis-related outcomes found no evidence supporting direct anti-fibrotic properties of PZQ. A likely explanation, therefore, would be that multiple infection-treatment cycles may increase host sensitization to parasite antigens, potentially resulting in amplified type 2 immune responses that exacerbate inflammation and fibrosis from newly deposited eggs (60). In agreement with that, our previous study found that mice with more treatment/reinfection cycles showed partial protection (reduced egg burdens) (61) but comparable fibrosis levels per egg, suggesting that repeated exposure may increase anti-parasitic responses that on the one site would prevent further egg deposition but on the other side foster pro-fibrotic responses to any newly deposited egg hence persistent tissue pathology. Moreover, increased total IgE also supports the idea of immune priming, which is consistent with earlier research showing a link between total IgE and human resistance to reinfection (62, 63).

Mechanistically, this dichotomy of PZQ-treatment driven immune responses, both protective against reinfection and pro-fibrotic, could be linked to metabolomic changes, as children with more PZQ rounds showed heightened arginine-proline metabolism associated with type-2 immune responses. In fact, this pathway is reported to be both protective against reinfection and to foster collagen deposition driving tissue fibrosis (20, 30, 64–66), potentially conditioned at the epigenetic level by sustained exposure to parasite antigens through infection-treatment cycles. Yet again, such an observation of a more activated type-2 metabolomic and immunological arm of the host responses argue against the sole likelihood of just better PZQ- mediated parasite clearance in the SAC receiving more treatment rounds as supporting the reduced egg burden and ameliorated morbidity observed. A sole play of better clearance in some hosts when compared to less treated ones would have otherwise been materialized by a reduction of all parasite-directed responses (since type- 2 metabolomic responses are prompted by the parasite to actively drive processes like tissue fibrosis) in hosts with less parasite burden, which is not the case in our setting.

Of note, however, on the overall observations from our study, several limitations should be considered. Firstly, the observational design limits our capacity to establish a causal relationship between the number of PZQ treatment rounds and some study outcomes and would warrant more powered and longitudinal studies to confirm/inform the present report findings. Secondly, although, we observed an association between cumulative PZQ exposure and parasite burden, we did not directly evaluate the immunological mechanisms that might lead to reduced egg burden (anti-worm and/or anti-fecundity (13)); adult worm quantification in the SAC (CCA or CAA-based) would have helped defined the direct impact on adult worm burden as well and start to disentangle the effects on worm viability versus worm fecundity for our observed outcome. Thirdly, the relatively small sample size limits the generalizability of our findings but provide just enough evidence to support our preclinical observations of an association of repeated treatment in endemic area with reduced likely of heavy infection thereafter, pending a definitive longitudinal study to assess the causal relationships existing here. In addition, on cognitive evaluation of SAC, a possible survivorship bias cannot be excluded, as children who remained in school and therefore received more annual PZQ rounds may have inherently better academic performance or school attendance. To minimize this bias, school attendance (assiduity) should be included and adjusted in variable in the analysis for future studies. Overall, future research would benefit from larger, longitudinal studies with extended follow-up to better understand the long-term immunological and pathological consequences of repeated PZQ administration in endemic settings. Analyses of antigen-specific antibody responses to different parasite preparations may also shed more light on the possible immunological patterns linked to repeated PZQ administration in endemic settings. Finally, our study site is also affected by other parasites that share pathological features with schistosomiasis such as malaria and filariasis, which were not specifically explored for the present study. Given likelihoods of cross-reactivity from these various parasites, follow-up investigative undertakings are warranted to understand the true dynamic under polyparasitic settings of the observed negative association between repeated cycles of infection/treatment with PZQ in schistosomiasis-exposed SAC and likelihood of heavy reinfection with such schistosomes.

**Figure 6:**
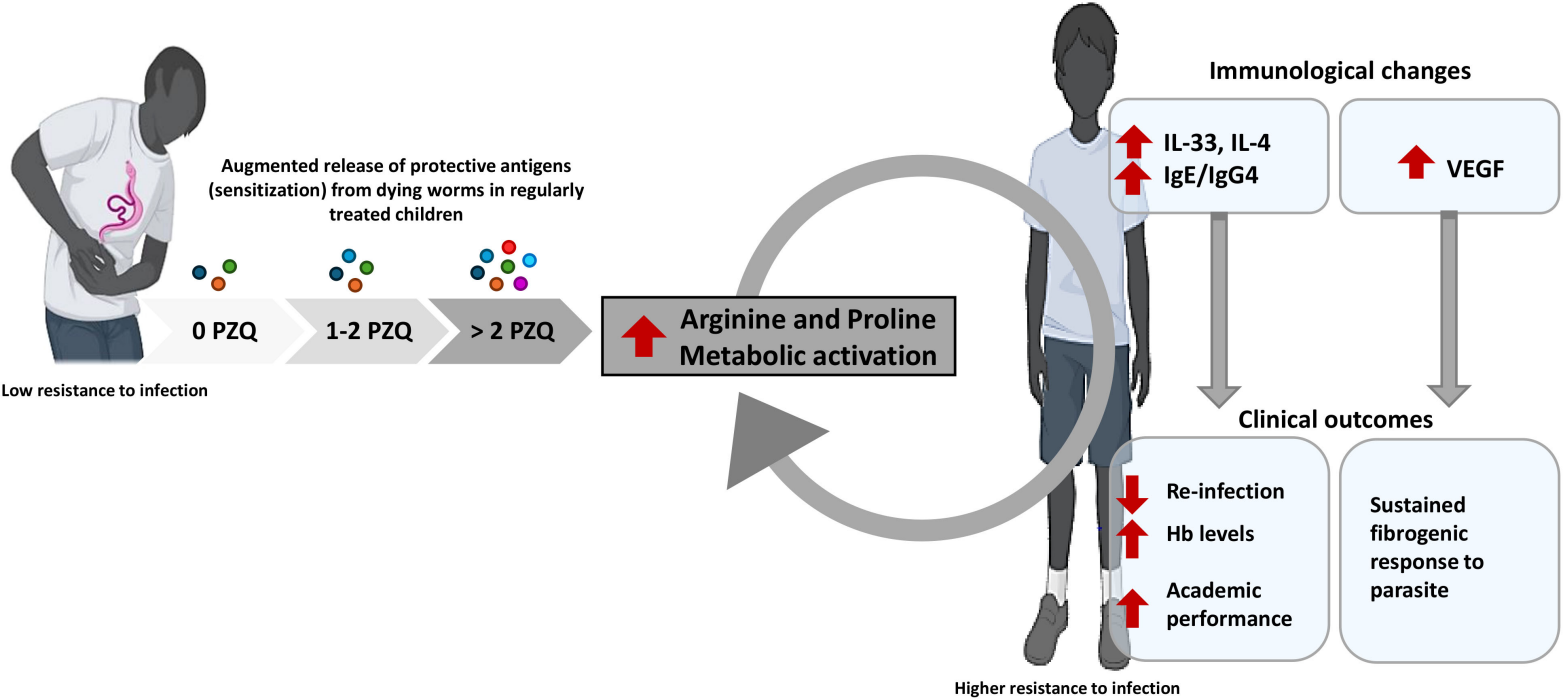
Model and interpretation of the observed clinical, cognitive, immunological and metabolomic changes in SAC from schistosomiasis endemic area with history of sustained MDA-based PZQ treatments. In response to increasing numbers of MDA OF PZQ in SAC from *S. mansoni* endemic area, parasite protective antigens might be increasingly shed fostering recognition by the host and prompting metabolomic changes such as increased arginine/proline metabolism, leading to higher production of several host protective soluble mediators (IL-33, IL-4 and IgE). This in turn drives protection against *S mansoni* reinfection, and limits significantly anemia to ameliorate the SAC wellbeing and cognitive performance. However, sustained arginine/proline metabolism pathognomonic of chronicity might also heighten the release of the angiogenic factor VEGF that aligns with a continued onset of liver fibrosis despite a paradoxically augmented resistance to infection in parallel. A case for bidirectional action of repeated cycles of PZQ treatment in SAC from endemic area is proposed whereby immunity to reinfection is acquired, as a result of accelerated exposure to host protective antigens from dying worms, at the expenses of sustained/elevated reactivity of the host against these parasite antigens that might support a continuous/elevated fibrogenicity of subsequent *S. mansoni* infection. Cartoons were generated using Biorender.

As of yet, our study provides support clinical evidence to our previous preclinical posit (28) that repeated PZQ administration is associated with enhanced host immunity to reinfection and improved disease outcomes (increased hemoglobin, enhanced neurocognitive performance). However, this protective immune profile might not be accompanied by antifibrotic activity as indicated by elevated VEGF and persistent liver fibrosis risk, suggesting a potential “double-edged sword” effect where immunity to PZQ-killed worm may inadvertently promote tissue fibrosis in infection-resistant hosts (**Figure 6**). Above all, these findings provide, exploratively, timely translational insights on the scope of action of PZQ-based MDA strategies for schistosomiasis elimination as a public health problem.

## Data Availability

The original contributions presented in the study are included in the article and SupplementaryFiles. The metabolomics data for this study are publicly available in the Metabolomics Workbench repository and can be accessed via the study summary search page (https://www.metabolomicsworkbench.org/data/DRCCStudySummary.php) using the DataTrack ID: 7105. Further inquiries can be directed to the corresponding author.

## Supplementary material

Supplementary Figure 1 Map of the study site. **(A)** Localization of Cameroon in Africa. **(B)** Localization of the Centre region in Cameroon. **(C)** Position of the Mbam and Inoubou department within the Centre region of Cameroon. **(D)** Bokito subdivision and Study area.

Supplementary Figure 2 Correlation of cytokine and antibody levels with infection burden. **(A)** Relationship between plasma levels of IL-4 and infection burden. **(B)** Relationship between plasma levels of IL-33 and infection burden. **(C)** Relationship between plasma levels of IgG4 and infection burden. Statistical analyses: Spearman correlation with correlation coefficient (r) and p-value (p) reported. Infection burden is expressed as Eggs per gram of stool (EPG).
