# Supplementary figures and images for: Mass drug administration of praziquantel lowers the susceptibility of school-aged children to Schistosoma mansoni in endemic areas
Source: Front Immunol. 2026 Feb 25;17:1746003. doi: 10.3389/fimmu.2026.1746003 (PMC12975766; doi:10.3389/fimmu.2026.1746003)

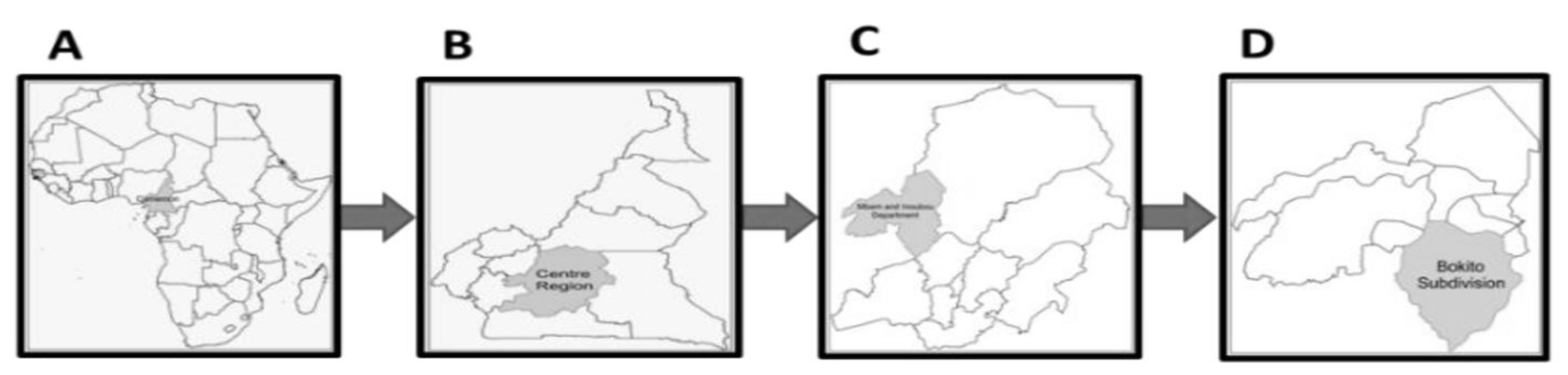

Supplement: Supplementary Figure 1 — Map of the study site. (A) Localization of Cameroon in Africa. (B) Localization of the Centre region in Cameroon. (C) Position of the Mbam and Inoubou department within the Centre region of Cameroon. (D) Bokito subdivision and Study area. [file Image1.tif]

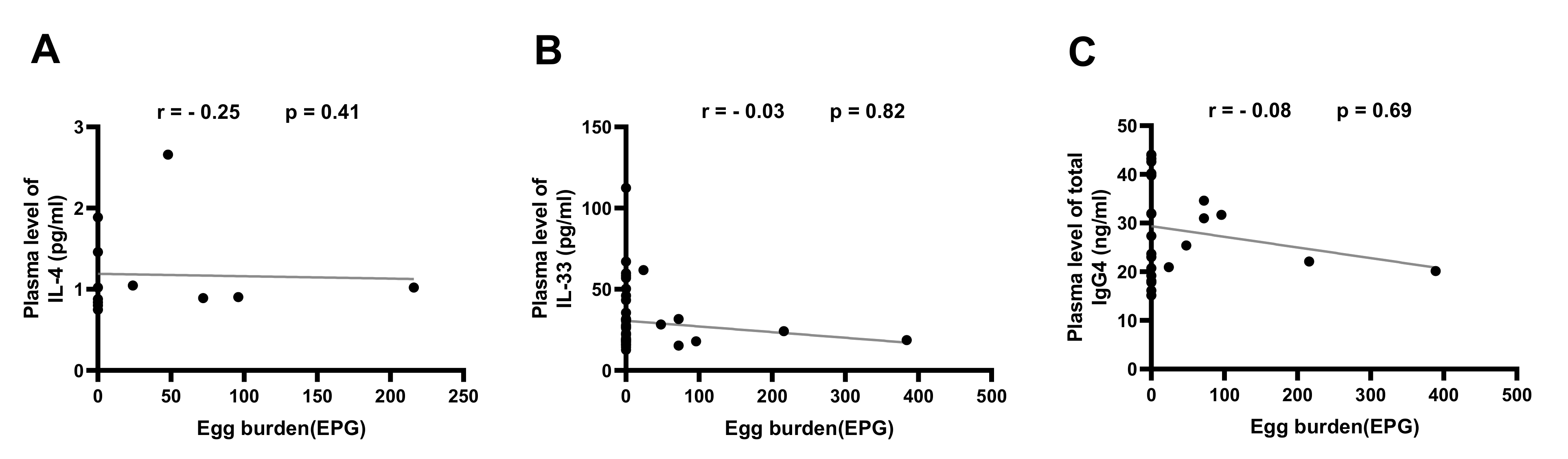

Supplement: Supplementary Figure 2 — Correlation of cytokine and antibody levels with infection burden. (A) Relationship between plasma levels of IL-4 and infection burden. (B) Relationship between plasma levels of IL-33 and infection burden. (C) Relationship between plasma levels of IgG4 and infection burden. Statistical analyses: Spearman correlation with correlation coefficient (r) and p-value (p) reported. Infection burden is expressed as Eggs per gram of stool (EPG). [file Image2.tif]
